# Immunohistochemical angiogenic biomarkers in hepatocellular carcinoma and cirrhosis: correlation with pathological features

**DOI:** 10.6061/clinics/2016(11)04

**Published:** 2016-11

**Authors:** Osmar Damasceno Ribeiro, Nathalie Henriques Silva Canedo, Vera Lucia Pannain

**Affiliations:** I Universidade Federal do Rio de Janeiro, Programa de Pós-Graduação em Anatomia-Patológica, Rio de Janeiro/RJ, Brazil; II Universidade do Oeste de Santa Catarina (UNOESC), Faculdade de Medicina, Joaçaba/SC, Brazil; III Universidade Federal do Rio de Janeiro, Faculdade de Medicina, Departamento de Patologia, Rio de Janeiro/RJ, Brazil

**Keywords:** VEGF, CD105, COX-2, Hepatocellular Carcinoma, Cirrhosis, Pathology

## Abstract

**OBJECTIVE:**

To investigate immunohistochemical markers of angiogenesis and their association with pathological prognostic features in hepatocellular carcinoma and cirrhotic liver.

**METHODS:**

Vascular endothelial growth factor, CD105, and cyclooxygenase-2 were immunohistochemically detected in 52 hepatocellular carcinoma tissue samples and 48 cirrhotic liver tissue samples. Semiquantitative measurements of vascular endothelial growth factor and cyclooxygenase-2 were evaluated considering the degree and intensity of immunostaining based on a 7-point final scoring scale. CD105 microvascular density (MVD-CD105) was measured using automated analysis. Morphological aspects evaluated in the hepatocellular carcinoma samples included size (≤2 and >2 cm), differentiation grade, and microvascular invasion.

**RESULTS:**

The mean vascular endothelial growth factor immunoreactivity score was slightly higher in the hepatocellular carcinoma samples (4.83±1.35) than the cirrhotic liver (4.38±1.28) samples. There was a significant and direct correlation between these mean scores (*r_s_*=0.645, *p*=0.0001). Cyclooxygenase-2 was expressed in all the cirrhotic liver samples but was only found in 78% of the hepatocellular carcinoma samples. The mean cyclooxygenase-2 score was higher in the cirrhotic liver samples (4.85±1.38) than the hepatocellular carcinoma samples (2.58±1.68), but there was no correlation between the scores (*r_s_*=0.177, *p*=0.23). The mean CD105 percentage in the hepatocellular carcinoma samples (11.2%) was lower than that in the cirrhotic samples (16.9%). There was an inverse relationship in MVD-CD105 expression between the hepatocellular carcinoma and cirrhotic samples (r_s_=-0.78, *p*=0.67). There were no significant associations between vascular endothelial growth factor expression and morphological characteristics. Cyclooxygenase-2 and CD105 were associated with hepatocellular carcinoma differentiation grade (*p*=0.003 and *p*=0.05, respectively).

**CONCLUSION:**

Vascular endothelial growth factor, cyclooxygenase-2, and MVD-CD105 were highly expressed in cirrhotic liver compared to hepatocellular carcinoma and might be involved in liver carcinogenesis. Additionally, cyclooxygenase-2 and CD105 might be involved in hepatocellular carcinoma differentiation grade.

## INTRODUCTION

Hepatocellular carcinoma (HCC) is a peculiar neoplasm with characteristics that differ from other tumor types, the major difference being that it most often occurs in association with chronic liver disease.

Gene expression studies in tumorous and surrounding non-tumorous liver tissues have identified molecular profiles associated with tumor differentiation, recurrence, vascular invasion, and patient survival 1-3. Angiogenesis activation pathways have been reported to play a role in HCC carcinogenesis 4.

Vascular endothelial growth factor (VEGF) is one of the most important factors involved in tumoral angiogenesis. VEGF is a potent endothelial cell mitogen that induces the formation of new vessels and increases vascular permeability 5. In addition, VEGF expression in HCC has been correlated with tumor aggressiveness (capsular infiltration, vascular invasion and intrahepatic metastasis) 6-8 and microvascular density (MVD) 9.

Immunohistochemical evaluation of MVD using antibodies against CD34, CD31, and CD105 has been performed to evaluate tumor angiogenesis. However, CD105 is a more specific marker for tumor angiogenesis than CD34 or CD31, which are pan-endothelial markers 10.

Cyclooxygenase-2 (COX-2) participates in carcinogenesis via different mechanisms, such as by promoting angiogenesis and cellular proliferation and inhibiting apoptosis 11. High COX-2 expression has been demonstrated in non-HCC tumors, including gastrointestinal cancers 12-14. In addition, COX-2 15,17, VEGF 6,16 and CD105 17 are expressed in chronic liver diseases.

Considering that VEGF, COX-2, and CD105 are involved in tumoral angiogenesis and that HCC is a highly vascularized tumor that occurs mainly in chronically diseased livers exhibiting neoangiogenesis, we investigated immunohistochemical expression patterns of VEGF, COX-2, and MVD-CD105 in HCC and surrounding cirrhotic liver tissues. Furthermore, we evaluated the putative association of these markers with HCC pathological features, including tumor size, differentiation grade, and microvascular invasion status.

## MATERIAL AND METHODS

### Patients and pathology findings

We studied 52 HCC and 48 surrounding cirrhotic liver tissue samples obtained during orthotopic liver transplantation or partial hepatectomy procedures performed in 38 male and 14 female adult patients with cirrhosis (aged 18–69 years). Forty-three patients had hepatitis C virus infection in isolation or in association with other etiologies. Twenty-six tumors were ≤2 cm in diameter, and 26 were >2 cm in diameter. According to grading, 40 of the 52 HCC tumors were well differentiated (grade I and II), 7 were moderately differentiated (grade III), and 5 were poorly differentiated (grade IV). In cases with more than one histological grade, the highest grade was considered. Microvascular invasion was present in 23 (44.2%) tumors.

This study was approved by the local ethics committee of our institution (N° 163/11)

### Immunohistochemistry

Paraffin-embedded tissues were sectioned. The sections (5 µm) were deparaffinized in xylene, dehydrated and rehydrated in ethanol, and subjected to steamer antigen retrieval. The sections were then processed using a Novocastra Novolink polymer detection system (Leica Biosystems Newcastle Ltd, UK) with the following primary antibodies: mouse anti-human VEGF C-1 (sc-7269, 1:6000; Santa Cruz Biotechnology Inc, USA), mouse anti-human CD105 (Endoglin) (clone 4G11, 1:60; Leica Biosystems Newcastle Ltd, UK), and rabbit anti-human COX-2 (clone SP21,SPB-M3212, 1:500; Spring Bioscience, USA). Negative controls lacked the primary antibody and were processed simultaneously.

### Evaluation of immunohistochemical staining

VEGF and COX-2 immunostaining was scored semiquantitatively according to modification of a previous method 18. Briefly, positive immunostaining was assessed in tumor cells for the HCC samples or hepatocytes for the cirrhotic tissues. Staining intensity was scored as 0, negative; 1, weak; 2, moderate; or 3, strong. The degree of staining was scored based on the percentage of positive cells as follows: 0, 0–4.9%; 1, 5–25%; 2, 26–50%; 3, 51–75%; and 4, 76–100%. When there was any doubt in the score to be assigned, the highest score was chosen. The sum of the intensity and the degree of the staining scores was used to produce a final overall score as follows: 0–1, negative; 2–3, weak; 4–5, moderate; and 6–7, strong. Lastly, the final mean scores were evaluated for all tissues.

CD105-stained tissues were evaluated using computer-based quantitative analysis to assess MVD. For the analyses, 3 microscope fields showing the highest staining intensity (hot spots), such as blood vessels or endothelial cells, were examined using an Olympus microscope, model BX-40. All images were captured using an Olympus digital 3.3-megapixel camera attached to the microscope. The images were analyzed using the “color function” and area/density measurement function in Imagelab® software. The MVD-CD105 percentages were calculated over a fixed area of 216 μm for each field. The average of the 3 fields was recorded as the mean MVD score.

### Statistical analysis

Fisher’s exact test or the *χ*^2^ test was used to compare categorical variables. The Mann-Whitney test or Spearman's rank (rs) correlation coefficient was used to compare continuous variables. Quantitative data are presented as the mean±standard deviation (SD). Statistical analyses were performed using SAS^®^ System software, version 6.11 (SAS Institute, Inc., Cary, North Carolina), and *p*<0.05 was considered statistically significant. The statistical methods used in this study were reviewed by a biomedical statistician from the Division of Biostatistics at Clementino Fraga Filho Hospital.

## RESULTS

### VEGF, COX-2, and MVD-CD105 expression in HCC samples and surrounding cirrhotic liver tissues

**Figure 1:**
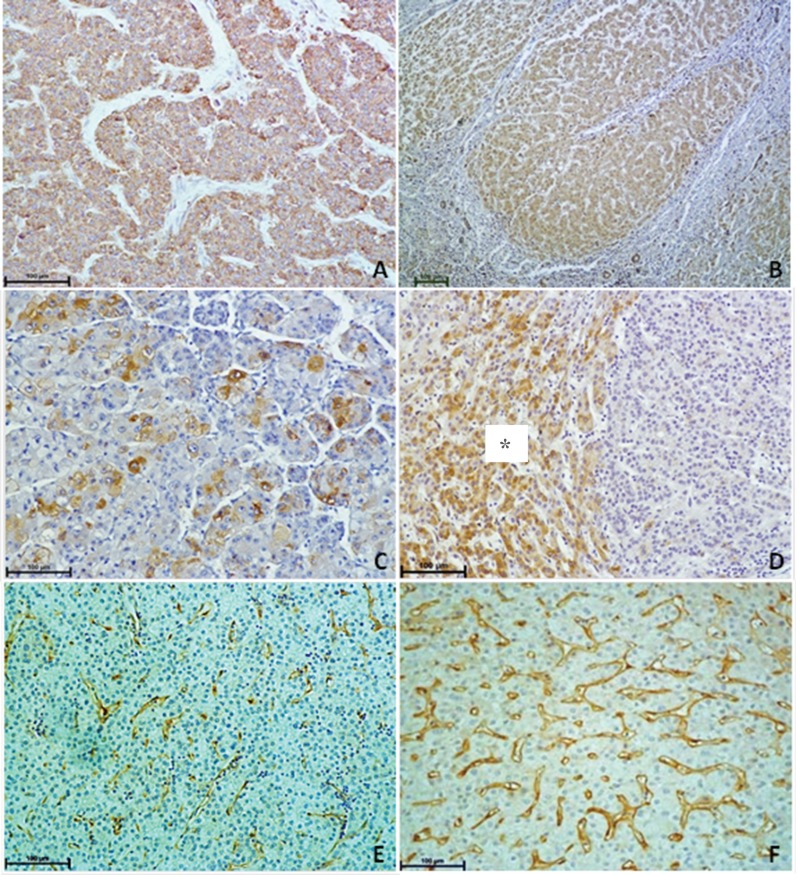
Immunohistochemical staining in HCC and cirrhotic tissues. Immunohistochemical staining in HCC samples: VEGF (A), COX-2 (C), and CD-105 (E). Immunohistochemical staining in cirrhotic liver samples: VEGF (B), * refers to the area of expression of COX-2 (D), and CD-105 (F).

Diffuse cytoplasmic VEGF expression was detected in all HCC and cirrhotic liver tissue samples. The mean VEGF score was slightly higher in the HCC tissues (4.83±1.35) compared with the cirrhotic tissues (4.38±1.28). There was a significant and direct association between VEGF expression in the HCC and cirrhotic tissues (*r_s_*=0.645, *p*=0.0001). COX-2 was expressed in all the cirrhotic liver samples but only in 78% of the HCC samples. The mean COX-2 score was higher in the cirrhotic tissues (4.85±1.38) compared with the HCC tissues (2.58±1.68), but there was no association between the two (*r_s_*=0.177, *p*=0.23). The mean MVD-CD105 values were lower in all HCC samples (11.2%) compared to cirrhotic tissues (16.9%). Although there was no significant correlation in MVD-CD105 percentages between the HCC and cirrhotic tissues, an inverse relationship was apparent (r_s_=-0.78, *p*=0.67). Examples of immunohistochemical staining in the HCC and cirrhotic tissues are shown in Figure 1.

### Correlation of VEGF, COX-2, and MVD-CD105 with pathological features

In the HCC cases, cytoplasmic VEGF expression in tumor cells was mostly moderate or strong (48.32% or 36.53%, respectively). Although expression intensity did not significantly differ in relation to tumor size (*p*=0.37), a moderate expression pattern predominated in smaller tumors (<2 cm). There was no association between VEGF expression and HCC differentiation grade or microvascular invasion status, although moderate-intensity staining predominated in well-differentiated HCC (Table 1). COX-2 expression was significantly associated with HCC differentiation grade (*p*=0.003), but not with size or microvascular invasion status (Table 2). Likewise, the mean MVD-CD105 values were not associated with microvascular invasion status or tumor size in the HCC tissues, although the mean MVD-CD105 expression tended to be higher in smaller tumors (≤2 cm). However, MVD-CD105 expression was significantly higher in well-differentiated HCC tissues compared with poorly and moderately differentiated tissues (*p*=0.05) (Table 3). There were no correlations between the mean VEGF, COX-2, or MVD-105 scores. However, inverse relationships were noted between the MVD-105 and VEGF scores (*r_s_*=-0.13, *p*=0.33) and the MVD-105 and COX-2 scores (*r_s_*=-0.15, *p*=0.29).

## DISCUSSION

VEGF acts as a key mediator of tumoral angiogenesis by increasing vascular permeability and promoting endothelial proliferation and migration 19. In the liver carcinogenesis, the VEGF expression increases gradually from its early stages 20. VEGF expression has been associated with patient prognosis in terms of overall survival and recurrence 21, including after liver transplantation when observed in adjacent cirrhotic tissue 22.

Our data showed that mean VEGF expression was slightly higher in HCC tissues compared with surrounding cirrhotic tissues, and there was a positive correlation between them. This suggested a close relationship between VEGF expression and carcinogenesis, which was congruent with previous observations 23 and reinforces the importance of VEGF in HCC. Conversely, other studies have reported the predominance of VEGF in non-tumorous cirrhotic tissue 6,24, but not in cirrhotic nodules distant from HCC 25. It is possible that we observed higher VEGF scores in HCC because we evaluated smaller tumors (≤2 cm) than those examined in other studies (5 cm). In the present study, all HCC and surrounding cirrhotic tissues expressed VEGF, which is in agreement with the findings of Imura et al. 26, who observed VEGF expression in all HCC tissues. In contrast, in another study, VEGF positivity was reported in 66.7% of HCC samples and 79.4% of cirrhotic tissues 6. Disagreements between studies using immunohistochemical profiling might arise because of the different methods employed to score staining. However, in accordance with the same study 6, we did not observe any significant associations between VEGF expression and differentiation grade, microvascular invasion status, or tumor size. This might suggest that VEGF-stimulated angiogenesis does not change significantly during HCC progression.

In HCC, vascularization can result in the development of new vessels by neoangiogenesis or from the preexisting sinusoidal network by angiogenesis 27. MVD, assessed by CD105 immunostaining, has been suggested as a quantitative measure of tumoral angiogenesis and has been reported as a prognostic indicator of outcome in malignant tumors, including HCC 7. In the present study, MVD-CD105 expression was slightly higher in smaller tumors, although it was not significantly different compared to that in larger tumors (>2 cm). Similarly, MVD-CD105 expression was not associated with microvascular invasion status, but it was associated with well-differentiated HCC. Additionally, MVD-CD105 expression tended to decrease as grade increased. Ho et al. 28 found a correlation between low MVD-CD105 expression and a more aggressive tumor type, as indicated by a large size and microvascular invasion. It is possible that the lack of association between MVD-CD105 expression and microvascular invasion in the present study resulted from the smaller average size of the advanced lesions that were examined (4.8 cm); in contrast to our current work, Ho et al. compared tumors that were ≤5 cm in diameter with those >5 cm in diameter. Such findings in combination with our previous observations 29 suggest that MVD-CD105 expression decreases during carcinogenesis and tumor progression.

COX-2 overexpression has been documented in patients with HCC with concomitant hepatitis virus infection 14. Immunohistochemical staining revealed COX-2 expression in the majority of HCC tissues and all cirrhotic tissues evaluated in the present study, which was concordant with previous studies 15 17. Moreover, previous studies have indicated that COX-2 RNA expression is higher in adjacent liver tissues than in HCC tissues 30. In contrast, other reports have found 100% of HCC samples showing COX-2 positivity 31,32. Hepatitis C virus infection can promote COX-2 expression 33, which might explain why all the non-tumorous tissues in the present study were COX-2-positive. We found that COX-2 expression was associated with HCC differentiation grade, but not with microvascular invasion status or tumor size. COX-2 expression was higher in well-differentiated HCC, suggesting that it may be involved in the early stages of carcinogenesis 31,32. Cervello et al. 32 suggested that COX-2 overexpression in tumor progression might cause growth disadvantages in certain cells. Regarding tumor size, Koga et al. 31 observed a predominance of COX-2 in small HCC tumors compared with large tumors. Epigenetic studies have implied that reduced COX-2 expression is associated with poor prognosis in patients with HCC 30.

In conclusion, this study shows that VEGF, COX-2, and MVD-CD105 expression were higher in cirrhotic liver tissues compared with HCC tissues, which suggests that these proteins play important roles in liver carcinogenesis. In addition, both COX-2 and MVD-CD105 expression appeared to play roles in HCC differentiation.

## AUTHOR CONTRIBUTIONS

Ribeiro OD performed the research and drafted the manuscript. Canedo NH analyzed the data. Pannain VL designed the research and revised the manuscript. All authors read and approved the final version of the manuscript.

## Figures and Tables

**Table 1:** VEGF expression patterns according to morphological features.

Variables	n	VEGF expression (%)				*p*
		N	Weak	Mod	Strong	
**Size**						0.37
**≤2.0 cm**	26	-	15.4	57.7	26.9	
**>2.0 cm**	26	-	15.4	38.5	46.1	
**Differentiation**						0.91
**Grade I/II**	40	-	15.0	50.0	35.0	
**Grade III/IV**	12	-	16.7	41.7	41.6	
**MI invasion**						0.53
**Present**	23	-	17.4	39.1	43.5	
**Absent**	29	-	13.8	55.2	31.0	

N – negative; Mod – moderate; MI – microvascular

**Table 2:** Cox-2 expression patterns according to morphological features.

Variables	n	COX-2 expression (%)				*p*
		N	Weak	Mod	Strong	
**Size**						0.12
**≤2.0 cm**	26	15.4	57.6	23.1	3.9	
**>2.0 cm**	26	26.9	30.8	42.3	-	
**Differentiation**						0.003
**Grade I/II**	40	10.0	52.5	35.0	2.5	
**Grade III/IV**	12	58.3	16.7	25.0	-	
**MI invasion**						0.84
**Present**	23	26.1	39.1	34.8	-	
**Absent**	29	17.2	48.3	31.0	3.5	

N – negative; Mod – moderate; MI – microvascular

**Table 3:** Microvascular density of CD105 according to morphological features.

Variables	n	MDV-CD105		*p*
		Average	SD	
**Size**				0.1
**≤2.0 cm**	26	12.5	5.2	
**>2.0 cm**	26	9.92	4.43	
**Differentiation**				0.05
**Grade I/II**	40	11.9	5.0	
**Grade III/IV**	12	8.87	4.49	
**MI invasion**				0.12
**Present**	23	9.8	3.9	
**Absent**	29	12.31	5.48	

N – negative; SD – standard deviation; MI – microvascular; MDV – microvascular density
